# Antiplasmodial Activity of the Crude Extract and Solvent Fractions of Stem Barks of *Gardenia ternifolia* in *Plasmodium berghei*-Infected Mice

**DOI:** 10.1155/2021/9625169

**Published:** 2021-09-01

**Authors:** Dejen Nureye, Muktar Sano, Mesfin Fekadu, Tadesse Duguma, Eyob Tekalign

**Affiliations:** ^1^Department of Pharmacology and Toxicology, School of Pharmacy, College of Medicine and Health Sciences, Mizan-Tepi University, P.O. Box 260, Mizan-Aman, Ethiopia; ^2^Department of Pharmacy, College of Health Sciences, Arsi University, P.O. Box 193, Asella, Ethiopia; ^3^Department of Medical Laboratory Sciences, College of Medicine and Health Sciences, Mizan-Tepi University, P.O. Box 260, Mizan-Aman, Ethiopia

## Abstract

**Background:**

The evolution of resistance to currently used malaria medicines together with the severe economic burden of malaria initiates the search for novel antimalarial drugs. Thus, the present experiment was intended to assess the *in vivo* antiplasmodial effect of *Gardenia ternifolia* based on the traditional claims and in vitro antimalarial effect of the plant.

**Methods:**

For the crude extraction of stem barks of *G. ternifolia*, a cold maceration method using hydromethanol as a solvent was employed. The hydroalcoholic extract was then fractionated by three solvents (chloroform, n-butanol, and aqueous solvent) with different polarity indexes. Swiss albino mice infected with the chloroquine-sensitive malaria parasite (*Plasmodium berghei*) were used in this study. Acute oral toxicity study was done according to standard protocols. Four-day suppressive (hydromethanolic crude extract and solvent fractions), Rane's (crude extract), and repository (crude extract) tests were used to examine the antiplasmodial effects of the study plant.

**Results:**

The chemosuppressive study revealed that all doses of the crude extract and its fractions displayed a significant (*P* < 0.001) inhibition of parasitemia compared with the vehicle (negative control). The crude extract's highest dose (600 mg/kg) showed the maximum (57.84%) parasitemia suppression during the chemosuppressive test. The crude extract also produced significant (*P* < 0.001) curative and prophylactic effects at all doses in Rane's and repository tests compared with the negative control. In the 4-day test, the n-butanol fraction produced parasitemia suppression higher than the chloroform fraction but lower than the crude extract. Of these, water fractions demonstrated the lowest chemosuppressive effect. Anthraquinone, alkaloids, flavonoids, saponins, steroids, tannins, and terpenoids were qualitatively detected in the plant material.

**Conclusion:**

The current results showed that the hydromethanolic extract and fractions of *G. ternifolia* stem barks have antiplasmodial action with a high curative effect. Chloroform and n-butanol fractions were more active among the fractions, indicating that the nonpolar and semipolar constituents of the plant are responsible for the antimalarial effects.

## 1. Introduction

Malaria is a mosquito-borne disease caused by *Plasmodium* transmitted by the bite of female *Anopheles* mosquito. It continues to be the main health problem in most countries of the world. Malaria is still one of the main causes of morbidity and mortality in tropical and subtropical areas of the world. In addition to health impacts, malaria has produced a severe economic burden in many developing countries including Africa [1]. Five of the human pathogens—*Plasmodium falciparum*, *P. vivax*, *P. malariae*, *P. ovale* curtisi, and *P. ovale* wallikeri—are well-recognized etiologic factors for human malaria. Infrequently, we could be naturally or accidentally infected by many simian species such as *P. knowlesi*, *P. falciparum*, and *P. vivax* that create a huge challenge on public wellbeing. *P. falciparum* is most common in Africa, and the majority of deaths due to malaria are caused by this species [2]. From above 400 different *Anopheles* mosquito species, around 30 are vectors of major importance for malaria transmission [3].

Approximately 229 million cases of malaria, most (94%) from the African region, took place in 2019 globally. Malaria caused 409 thousand deaths worldwide and most (94%) of which are also from Africa. In 2019, the global case incidence and mortality rate of malaria was reduced by 57 and 10%, respectively. Nonetheless, malaria continued to hit children and pregnant mothers in Africa. Children aged <5 years are the most exposed group of the population affected by malaria (67% of global malaria deaths in 2019) [3]. Malaria is the most common infectious disease in Ethiopia [4, 5] About 30,485,416 Ethiopians are living at high-risk places to infection by malaria. In 2019, 213 deaths and 904,496 confirmed cases due to malaria were reported by the Ministry of Health (FMoH) from Ethiopia [3]. In spite of the reduced rate of malaria morbidity and mortality in Ethiopia since 2010 [3], a high occurrence of malaria was reported from certain regions in contrast to increased preventive measures coverage at the family level [6, 7].

Apart from reduction in incidence and prevalence, the transmission of malaria is continued throughout the globe. Hence, its control needs a combined approach comprising chemotherapy. However, the occurrence of resistant strains against commonly used antimalarial drugs is a major problem [8]. Despite the widespread development of resistance and difficulties in poor areas to afford and access effective antimalarial agents, currently used medicines (artemether, chloroquine, and quinine) are discovered from medicinal plants. So, it is essential to focus on traditionally claimed plants for inventing new antimalarial compounds for the future.

According to the World Health Organization (WHO), about 65% of the world population has incorporated medicinal agents in their primary health care modalities [9]. In many undeveloped countries, 70–95% of citizens used traditional medicine as a primary source of health care. For example, in sub-Saharan Africa, 85% of the population goes to traditional healers [10]. In Nigeria, Ghana, Mali, and Zambia, herbal remedies are prescribed at home as first-aid therapy for sixty percent of children with high malaria fever [11]. More or less, 80% of the population in Ethiopia is dependent on the locally prepared remedy that fundamentally involves medicinal plants [12]. Using traditional remedies to treat a wide range of illnesses including malaria is a common practice in Ethiopia. The country is prosperous in a variety of incredible plant biodiversity. Research done on various traditionally used Ethiopian medical plants confirmed their antiplasmodial activities. Some of these, among others, are *Moringa oleifera* [13], *Salvadora persica*, *Balanites rotundifolia* [14], *Euphorbia abyssinica* [15], *Nuxia congesta* [16], *Myrica salicifolia* [17], *Terminalia brownii* [18], *Hypoestes forskalei* [19], *Schinus molle* [20], *Bersama abyssinica* [21] and *Kniphofia foliosa* [22].

*Gardenia ternifolia* Schumach. & Thonn.—commonly known as “*Gambilo*” in Amharic [23], “*Kambeelloo*” in Oromifa [24], “*Kota*” in Gumuz [25], “*Gambela*” in Sidama [26], “*Shigidida*” in Gedeo [27], “*Duwong*” in Anuak [28], “*Brmaiyta*” in Konso [29] and “Bodut” in Meinit [30]—is a plant belonging to the family of Rubiaceae. It is a small tree or shrub used as a traditional remedy in tropical Africa. The leaves are in whorls of 3 on short, rigid side branchlets, roughly hairy on both surfaces, but have ovoid fruits; whereas the flowers are white, turning yellow when fading, sweetly scented, and solitary at the ends of the branchlets. Its flowering time is from September to December. The worldwide distribution is from Limpopo, South Africa, to Kenya and West Africa. The common habitats are wooded grassland, along streams, and termite mounds [31].

Different parts of *G. ternifolia* are ethnobotanicals used in Africa to manage respiratory infections, sore eyes, headache, migraine, hypertension, diabetes, gastrointestinal disorders, erectile dysfunction, malaria, convulsions, and epilepsy [32]. Moreover, its leaves are used to treat syphilis, skin diseases, and arrow poisoning [33, 34]. The stem part is used to arrest vomiting [35]. Barks have ethnomedical application for leprosy, ascites, hepatitis, onchocerciasis, female infertility, wound, and sexually transmitted diseases [36, 37]. The root is supposed to have antirheumatismal [38], antipain, antikwashiorkor [39], anticonstipation, anthelmintic, diuretic, and antirickets activities [40]. Alkaloids, flavonoids, phenols, saponins, tannins, terpenoids, steroids, quinones, stereoisomeric neolignans, and anthocyanins are the secondary metabolites detected from this species. Ethnopharmacological investigations revealed that the plant has antimicrobial, anti-inflammatory, cytotoxicity, antileishmanial, antioxidant, antimalarial, antisickling, antitheilerial, larvicidal, and hepatoprotective activities [32].

In Ethiopia, *G. ternifolia* is used medicinally by the tribal healer to treat hemorrhoid lesions (fruits) [41], the evil eye (roots and stem barks) [42], leg paralysis (stem barks), tooth bleeding (stem barks) [25], weight loss in children (barks), stomachache in livestock (barks) [26], and ulcerative lymphangitis in veterinary medicine (leaves) [27]. Additionally, barks of *G. ternifolia* roots and stems are reported as a traditional medicine to treat malaria in Ethiopia [24, 30]. Its stem barks, root barks, leaves, and leaf exudates have *in vitro* antimalarial activity against falciparum malaria [43, 44]. Hence, this experiment was intended to assess the antiplasmodial activity of the hydromethanolic extract and fractions of stem barks of *G. ternifolia* in rodent malaria models based on the previous ethnobotanical and ethnopharmacological studies.

## 2. Methods and Materials

### 2.1. Study Design

An experimental research method was used. To group and assign experimental animals for treatment, a technique called simple random sampling was used.

### 2.2. Chemicals, Reagents, and Materials

Absolute methanol, chloroform, n-butanol, distilled water, normal saline, trisodium citrate, oil immersion, Giemsa stain, chloroquine phosphate, 1 ml insulin syringes with needles, scissors, feeding tubes, electronic balances, gloves, and light microscopes were used in this study.

### 2.3. Plant Materials

The fresh *G. ternifolia* stem barks were collected in February 2020 from Bachuma Woreda, West Omo Zone, Southwest Ethiopia. The natural habitat of the plant is located about 581 kilometers from Addis Ababa. The samples were then evaluated and authenticated in Addis Ababa University, College of Natural and Computational Sciences, National Herbarium, where the certified sample number (ET 004/2020) was stored for future reference.

### 2.4. Experimental Mice

Both sexes of healthy Swiss albino mice (male for the study and female for acute toxicity) weighing 23–33 g, aged 6–8 weeks, were procured from Ethiopian Public Health Institute, Addis Ababa, and kept in the animal house at Mizan-Tepi University. The animals were left for a week for the purpose of acclimatization, and they were housed at room temperature in a stainless steel cage under a 12-hour light-dark cycle and provided water and commercial food pellets. All protocols and procedures employed in this study were in agreement with the National Institute of Health Guidelines for the Care and Use of Laboratory Animals [45]. Moreover, the protocol was accepted by the Committee of Research and Ethics, School of Pharmacy, Mizan-Tepi University.

### 2.5. Inoculum Parasite Species

*Plasmodium berghei* (ANKA) parasites that are sensitive to chloroquine were received from Ethiopian Public Health Institute to use in this study. The *Plasmodium* was preserved by serial passage of infected blood from infected to noninfected mice once in a weekly basis.

### 2.6. Plant Extraction and Fractionation

Thoroughly washed and cleaned fresh stem barks of the plant was chopped into pieces, air-dried under a shaded area with 20–25°C room temperature for 2 weeks, and then grounded into a coarse powder using a mortar and pestle. The dried powder (300 g) was weighed using a sensitive digital balance and soaked into 80% methanol (1000 ml) for three consecutive overnights. The process of extraction was facilitated by shaking occasionally (120 revolutions per minute) using a mechanical shaker (Bibby Scientific Limited, Stone, Staffordshire, UK). The plant residue was separated from the soaked plant extract by using sterile gauze and filtered in advance with Whatman No. 1 filter paper (Whatman®, England). This process was then repeated twice by adding another fresh solvent. The filtrate was collected, and the methanol was evaporated using a rotary evaporator device (Buchi Rotavapor R-200; Flawil, Switzerland) under a reduced pressure in a distillation flask at 45 revolutions per minute to obtain the crude extract. The extract was then dried and more concentrated in a drying oven at a temperature not exceeding 40°C. Finally, the filtrate (54 g (18% w/w)) was transferred to brown dishes (air-tight container) and firmly covered with aluminum paper and kept in a refrigerator at −20°C until used. By using three solvents having different polarities (chloroform, n-butanol, and water), 34 g of the crude hydroalcoholic extract was further fractionated. For fraction, the 80% methanolic extract was suspended in pure (distilled) water under a separator funnel, and then the suspension was mixed and shaken with the chloroform added. As a result, two layers were formed and a chloroform fraction was taken by separation. This step continues until the chloroform layer becomes clear. Then, the water layer was mixed with n-butanol and shaken to get butanol fraction. The n-butanol fraction was concentrated in a rotary evaporator, while the chloroform and the remaining fraction containing water are dried in a drying oven (Okhla Industrial Area, India) at no more than 40°C. Finally, the fractions packed in amber glass bottles were stored in a deep freezer (−20°C). The % (percentage) yield of chloroform, n-butanol, and water (aqueous) fractions was 9.64 (28.35%), 10.76 (31.65%), and 13.6 (40%), respectively.

### 2.7. Acute Oral Toxicity Test

Test for acute oral toxicity was conducted on the hydromethanolic extract and its fractions as per the Organization for Economic Co-operation and Development (OECD) guideline 425 [46]. Nonpregnant female Swiss albino mice that were fasted for 3 hours were used for toxicity studies. After the fasting time, the mice were weighed and a 2,000 mg/kg dose of crude extract and fractions were given through oral gavage, and then animals fasted for 2 hours. The first female mouse was followed up continuously for the first 30 minutes and then intermittently for every 4 hours within 24 hours. Since no behavioral changes and death were observed, another four female mice kept under the same conditions were administered the same dose, and signs of intoxication were observed for the next 14 days.

### 2.8. Mice Grouping and Dosing

Based on the pilot study and acute toxicity test results, 200, 400, and 600 mg/kg were determined to be used as doses for the 80% methanol extract and its fractions. In the experiment, 30 mice were randomly grouped into 5 (three treatment groups and two control groups), with six mice in each group. The negative control was treated by the solvents used in the reconstitution of the extracts (2% Tween-80 for chloroform fraction and 10 ml/kg pure/distilled water for the crude extract, n-butanol, and aqueous fractions). The positive control was treated with 25 mg/kg of chloroquine. The 3 treatment groups received 200, 400, and 600 mg/kg of either the crude extract or solvent fractions. The doses were given orally in a volume calculated as 10 ml/kg to each treatment group mouse.

### 2.9. Inoculation Preparation

The mice infected by *P. berghei* and with different levels of parasitemia were used as donor mice. The donor mice's parasitemia level was first determined from their blood that is obtained by cutting (0.5 to 1 mm section) the tail of the mice using scissors [47]. To inoculate and infect the study animals, the donor mouse with a parasitemia of 30 up to 37% [48] was sacrificed by a head blown technique, and blood was drained into a test tube containing anticoagulant (3.8% trisodium citrate (BDH Chemicals, England)) through the incision of the jugular vein. The collected blood was then diluted in normal saline to obtain 1 × 10^7^ infected red blood cells (RBCs) in every 0.2 ml suspension [49]. The dilution was done based on the erythrocyte count of the normal mice and parasitemia of the donor mice in such a way that 1 ml blood contains 5 × 10^7^ infected RBCs [47, 50]. Therefore, each mouse used was infected by 0.2 ml *P*. *berghei*-infected blood (1 × 10^7^ parasitized RBCs) intraperitoneally.

### 2.10. Early Infection (4-Day Suppressive) Test

Evaluations of antiplasmodial activity of the crude hydroalcoholic extract and its solvent fractions in early infection were performed according to the technique described by Peter's 4-day suppressive test [51]. Thirty male mice were infected and randomly assigned into five groups with six mice for each. All groups were treated for 2 hours of postinfection according to the methods mentioned above (mice grouping and dosing section). Treatment continues until day 4 (D3). On the 5^th^ day (D4), blood was collected from each mouse tail using clean, nongreasy, and labeled frosted slides and smeared using a spreader to make thin films. Air-dried thin films were then fixed with few drops of absolute methanol, left for approximately 10–15 minutes to air-dry, and stained for 15 minutes with 10% Giemsa stain at a pH of 7.2. The stain was washed off from the slides, and the slides were left to air-dry. The dried slides were then viewed through the light microscope using the oil immersion, and parasitemia was examined microscopically using the 100× objective. The parasitized RBCs were noted by the intracellular presence of the *Plasmodium* parasite. The parasitemia suppression percentage was calculated for each administered dose by comparing the parasitemia densities in infected control mice with those of treated mice in six randomly selected fields of the microscope. Each mouse's percentage parasitemia was determined on day 5 (D4), while mice body weight in g, rectal temperature in °C, and packed cell volume (PCV) in % were reported just before the infection at day 1 (D0) and at day 5 (D4).

### 2.11. Rane's (Curative) Test

Examination of the curative potential of the crude extract of the plant was done according to the procedure described by Ryley and Peters [52]. Thirty mice were selected and intraperitoneally injected with standard inoculum on D0 (first day). After seventy-two hours, the mice were randomly divided into five groups (*n* = 6) and provided treatment as described in the animal dosing and grouping section. Treatment continues every 12 hours until day 7 (D6). The blood was collected from the tail of each mouse, and thin films were made from day 3 (D3) up to day 7 (D7) to determine parasitemia levels. Body weight, rectal temperature, and PCV were recorded for each mouse just before the first dose and at the end of the experiment.

### 2.12. Prophylactic (Residual Infection) Test

The prophylactic activity of the crude extract was tested using the repository technique described by Peter's prophylactic test [53]. Weighed adult male mice were randomized into 5 of 6 mice each and treated with the respective dose for 4 days (D0–D3). On the 5^th^ day (D4), all mice were infected with the *Plasmodium* and followed up for 72 hours. Later, the parasitemia level was recorded. Measured data on body weight, rectal temperature, and PCV of the study mice were documented just before inoculation and at the end of the experiment.

### 2.13. Packed Cell Volume Determination

Blood was drawn from the grouped mice tail using capillary tubes (heparinized) to be filled ¾^th^ and sealed at the dry end with sealant. The capillary tubes were then placed in a hematocrit centrifuge, labeled according to grouped mice on PCV report format, centrifuged for five minutes at 12,000 revolutions per minute, measured, and calculated as follows [16]:

(1) $$\begin{array}{c} \text{PCV}=\frac{\text{volume of RBCs in a given volume of blood}}{\text{total volume of blood examined}}\times 100. \end{array}$$

### 2.14. Parasitemia Determination

Tail blood was dropped onto two labeled frosted microscope slides for each mouse, and then a thin smear was made and stained. Six arbitrarily selected fields on each slide were seen under a light microscope, and then the percentage parasitemia (PP) was determined as follows [19]:

(2) $$\begin{array}{c} \text{PP}=\frac{\text{number of Parasitized RBC}}{\text{total number of RBC}}\times 100. \end{array}$$

The hydromethanolic extract and its solvent fractions were compared with the controls in terms of parasitemia suppression. The formula below was used to measure the percent parasitemia suppression:

(3) $$\begin{array}{c} \begin{array}{c} \text{percentage parasitemia suppression}=\frac{\text{parasitemia in negative control}-\text{parasitemia in the treatment group}}{\text{parasitemia in negative control}}\times 100 \end{array}. \end{array}$$

### 2.15. Mean Survival Days

The study animals were supervised daily, and the number of days from the inoculation time up to death was documented for each mouse in control and treatment categories during the follow-up period at all models. Mean survival time (MST) for each group was decided by calculating the mean survival days of mice from the infection date over a period of 28 days using the formula described below [19]:

(4) $$\begin{array}{c} \text{MST}=\frac{\sum \text{ of survival time of all mice in a group }\left(\text{days}\right)}{\text{total number of mice in that group}}. \end{array}$$

### 2.16. Body Weight and Rectal Temperature

Each study mouse was weighed by means of a sensitive digital weighing balance. The rectal temperature of the mice was also measured by a digital rectal thermometer. The percentage changes of their mean results that occurred at pre- and post-treatment were then determined.

### 2.17. Phytoconstituent Analysis

The eighty percent methanolic crude extract and fractions of *G. ternifolia* stem barks were analyzed for the existence of secondary metabolites to correlate the antiplasmodial activity of the plant with these constituents [54, 55].

### 2.18. Quality Control

All materials used were of analytical grade. Data quality was maintained by randomization of the experimental mice during grouping, strict adherence to protocols, and coding of microscopic slides at the time of blood smear preparation. The animal attendants maintained the hygiene of the cages every 3 days by cleaning and removal of feces. Parasitized and noninfected RBCs were counted blindly by medical laboratory professionals.

### 2.19. Statistical Analysis

The data were entered in Excel 2010 software and exported to SPSS version 22 for analysis. The findings were presented as a mean ± standard error of the mean (SEM). Determination of statistical significance was carried out by one-way analysis of variance (ANOVA) and followed by comparison tests (Tukey's test) to compare the parameters (% parasitemia, % suppression, body weight, rectal temperature, and survival days) within and between groups. Additionally, the development of parasitemia across days of treatment in Rane's test was analyzed by two-way repeated-measures ANOVA. The analysis was executed with a 95% confidence interval, and *P* < 0.05 was taken as statistically significant.

## 3. Results

### 3.1. Acute Oral Toxicity Test

None of the test mice died or showed toxicity signs within 24 hours and the next 14 days after being treated by the hydroalcoholic extract and its fractions during acute oral toxicity study, indicating that the LD_50_ value of the study plant materials is more than 2,000 mg/kg.

### 3.2. Suppressive Activity of the Crude Extract and Solvent Fractions in the Four-Day Suppressive Test

The chemosuppressive study revealed that all doses of the hydroalcoholic extract and its fractions showed a significant (*P* < 0.001) inhibition of parasitemia compared with the vehicle agent (negative control) (Table 1). When the effect resulted from the doses of the crude extract was compared, the parasitemia suppression induced by the lower dose was significantly (*P* < 0.001) low in comparison with the effect produced by the higher dose. Similarly, significant variation (*P* < 0.01) was observed between 200 and 600 mg/kg of chloroform fraction. In the case of n-butanol fraction, inhibition of parasitemia by its lower dose was significantly (*P* < 0.001) lower than that of the 400 and 600 mg/kg doses. Comparable suppression was also seen between doses of the aqueous fraction. The n-butanol fraction produced parasitemia suppression higher than the chloroform fraction but lower than the crude extract. Of these, the water (aqueous) fraction displayed the lowest chemosuppression.

Besides, the 400 and 600 mg/kg doses of the hydroalcoholic extract were able to significantly (*P* < 0.001) increase survival days of treated experimental animals compared with the negative control. Likewise, both chloroform (*P* < 0.05 at middle dose, *P* < 0.001 at largest dose) and n-butanol (*P* < 0.001 at 400 and 600 mg/kg) fractions showed remarkable prolongation in survival date compared with the placebo treatment (Table 1). Nevertheless, the effect resulted by the test drugs in reducing parasitemia and prolonging MST was smaller than that of the standard drug.

Compared with the negative control (distilled water treatment), the crude extract protected body weight loss significantly (*P* < 0.05) at 600 mg/kg dose as shown in Table 2. The standard agent showed significant protection in body weight loss compared with the test drug (crude extract) and placebo agent (negative control). In the chloroform fraction-treated groups, there is no weight loss protection. However, the standard drug produced considerable prevention compared with the lower and middle doses of chloroform fraction as well as the negative control (2% Tween-80 treatment) with *P* < 0.01, *P* < 0.05, and *P* < 0.01 significance levels, respectively. N-butanol fraction averted significant reduction in body weight at 400 (*P* < 0.01) and 600 mg/kg (*P* < 0.001) doses when compared with the negative control. The prevention of loss in body weight by 600 mg/kg dose of the n-butanol fraction was comparable with the chloroquine (standard drug) effect. Comparison among doses of n-butanol fraction has been revealed that weight loss protective effect produced by the lowest dose was appreciably (*P* < 0.01) lower than that of the highest dose. In addition, the positive control was substantially elevated body weight in mice compared with the placebo therapy as well as the lower and middle doses of n-butanol fraction with significance levels *P* < 0.001, *P* < 0.001, and *P* < 0.01, respectively. Only the highest dose of aqueous fraction protected body weight loss in mice compared with the vehicle treatment (negative control). The effect shown by the three doses of the aqueous fraction was notably (*P* < 0.001) lower than the effect produced by the positive control (Table 2).

At the highest dose, the crude extract demonstrated a significant (*P* < 0.05) protective effect in rectal temperature drop in comparison with the negative control. Besides, the protective effect in rectal temperature decline by the hydromethanolic extract at the middle and highest doses was comparable with the effect of chloroquine (positive control). Chloroquine as well as the 400 and 600 mg/kg doses of all solvent fractions substantially (*P* < 0.05 to *P* < 0.001) averted the reduction in rectal temperature compared with the placebo treatment. Additionally, the 600 mg/kg of both chloroform and aqueous fraction exhibited a considerable (*P* < 0.01) preventive effect in comparison with the respective 200 mg/kg dose with regard to rectal temperature. Moreover, the protective effect in rectal temperature decrement by the three doses of all fractions was noticeably lower than the effect caused by the standard drug (Table 2).

**Figure 1:**
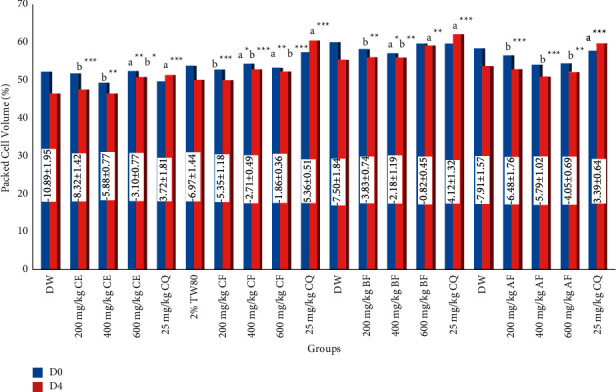
Packed cell volume of malaria-infected mice treated with crude extract and solvent fractions of *G. ternifolia* stem barks in the 4-day suppressive test. Values are presented as mean ± SEM (*n* = 6). ^*∗*^*P* < 0.05; ^*∗∗*^*P* < 0.01; ^*∗∗∗*^*P* < 0.001; a, compared with negative control; b, compared with positive control; d, compared with 400 mg/kg; e, 600 mg/kg; DW for distilled (pure) water (negative control); CE for crude extract; 2% TW80 for 2% Tween-80 (negative control); CF for chloroform fraction; BF for n-butanol fraction; AF for aqueous fraction; and CQ for chloroquine (positive control); D0, pre-treatment value on day 0; D4, post-treatment value on day 4.

The results of packed cell volume showed that the hydroalcoholic extract had a noteworthy (*P* < 0.01) preventive effect from anemia in mice compared with the placebo therapy (Figure 1). In both chloroform and n-butanol fractions, statistically significant protection of anemia was observed at 400 (*P* < 0.05) and 600 mg/kg (*P* < 0.01) when compared with the vehicle solvent (distilled water). Aqueous fraction did not protect reduction of PCV in mice. Nonetheless, with the exception of comparable effect by the 600 mg/kg dose of n-butanol fraction, the positive control exhibited substantial protective effect against anemia compared with groups treated by the hydromethanolic extract and all solvent fractions.

### 3.3. Curative Activity of 80% Methanolic Extract in Rane's Test

All doses of the 80% methanolic crude extract showed a considerable (*P* < 0.001) and dose-dependent curative effect compared with the negative control (Table 3). The extract also displayed statistically appreciably (*P* < 0.001) MST at 400 and 600 mg/kg when compared with the negative control. Statistically notable (*P* < 0.05 to *P* < 0.001) prolonged survival time was also found with regard to the comparison between extract doses. However, suppression of the parasite and an increase in survival time achieved by the crude extract was not more than those achieved by chloroquine.

**Figure 2:**
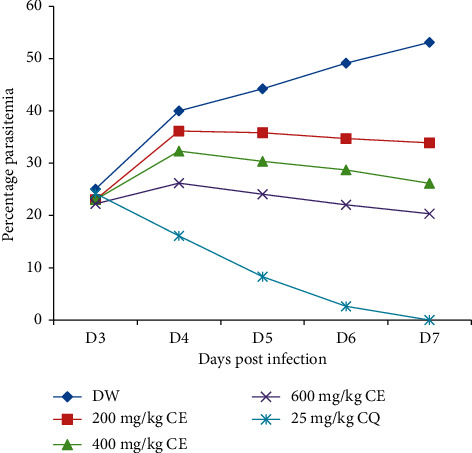
Parasitemia progress over the course of therapy with the crude extract of stem barks of *G. ternifolia* in Rane's test. Results are expressed as mean ± SEM (*n* = 6); DW for distilled (pure) water (negative control); CE for crude extract; and CQ for chloroquine.

Analysis of two-way repeated-measures ANOVA on parasitemia across treatment days showed a statistically important (*P* < 0.001) difference in the parasitemia level between the three doses of the crude extract and control groups (Figure 2). Observation of activity across days of treatment revealed that the parasitemia level was increased on day 4 (after the first dose) in the presence of distilled water and extract but decreased in the case of chloroquine-treated group. After the second dose administration, there was a gradual decrement of parasitemia during the course of crude extract therapy in all treated groups in contrast to the negative control. On day 7, the standard drug destroyed the parasite to an unnoticeable level (Figure 2).

The hydroalcoholic extract of stem barks of *G. ternifolia* had a protective effect with regard to reduction in body weight at 400 and 600 mg/kg doses (Table 4). A considerable (*P* < 0.01 to *P* < 0.001) protective effect was demonstrated by all doses of the extract in attenuation of rectal temperature compared with the solvent (negative control), although the effect by chloroquine was much higher. In addition, a substantial (*P* < 0.01) difference in the protection of rectal temperature reduction was noticed between the lowest and highest doses of the extract. Nevertheless, the extract did not attenuate PCV reduction in malaria-infected mice in the curative model.

### 3.4. Prophylactic Activity of the Hydroalcoholic Extract in the Repository Test

Chloroquine and all doses of the hydromethanolic extract suppressed parasitemia appreciably (*P* < 0.001) compared with the placebo drug (Table 5). Maximum suppression (*P* < 0.001) of parasitemia was achieved by the standard drug compared with all test doses, although complete eradication was not achieved. Comparison of parasite suppression among doses of the hydroalcoholic extract indicated that the lower dose had significantly low parasitemia suppression compared with the middle (*P* < 0.05) and higher (*P* < 0.001) doses. Survival time of the infected experimental mice pre-treated with the crude extract on the prophylactic study showed that 400 and 600 mg/kg doses were capable of prolonging survival time compared with the placebo treatment with *P* value <0.05 and 0.001, respectively. Comparison among doses of the extract showed that the 600 mg/kg dose notably (*P* < 0.01) prolongs MST compared with the lowest dose (Table 5).

Positive control and 600 mg/kg dose of the hydroalcoholic extract showed a remarkable (*P* < 0.001 and ^*∗∗∗*^*P* < 0.01, respectively) protective effect in body weight reduction compared with the negative control (Table 6). Although the effect was comparable with the highest dose, the standard agent drastically (*P* < 0.001) prevented body weight decline in comparison with the 200 and 400 mg/kg doses of the crude extract. Both chloroquine (*P* < 0.01) and the highest (*P* < 0.05) dose of the extract appreciably prevented rectal temperature reduction compared with the placebo agent. However, the standard drug had significant (*P* < 0.01) capacity compared with the 200 mg/kg dose of the hydromethanolic extract but produced a comparable effect compared with the middle and higher doses. Once more, a comparison between doses of the extract revealed that 600 mg/kg was better in improving the body weight and rectal temperature than 200 mg/kg as recorded in Table 6.

### 3.5. Phytoconstituent Screening

The preliminary phytoconstituent analysis of CE revealed the existence of all tested metabolites. Alkaloids, flavonoids, saponins, steroids, and tannins were noticed in both chloroform and n-butanol fractions, whereas anthraquinone and saponins were noticed in aqueous fractions (Table 7).

## 4. Discussion

Since malaria is the main community health threat in undeveloped countries and resistance occurred among currently used antimalarial agents, it is necessary to perform research toward the search for novel antimalarial compounds [56]. Concerning this, plants have been proved to be the main source in developing new antiplasmodial chemicals [57]. Therefore, studies should be done in screening claimed medicinal plants to provide potential lead compounds. The malaria parasites that cause human disease are basically not able to invade nonprimate animals. So, rodent malaria parasites were used for *in vivo* examination of antimalarial compounds [58]. The rodent malaria model has been fruitfully validated through screening many conventional antimalarial medicines [52]. The *in vivo* malaria model was also selected for the present experiment because it takes into account any pro-drug effect and the immune system activity in controlling infection compared with the *in vitro* experiment [50]. The *P. berghei* (ANKA) parasite was used in assessing the antiplasmodial activity of new chemicals in mice because all life cycle stages of the parasite are clearly seen on smears due to the nonadherence of the species with endothelial cells [47]. The four-day suppressive test used in the current study is the standard and widely used rodent malaria model for screening new antimalarial chemical entities [59]. In all chemosuppressive, Rane's, and repository tests, percentage parasitemia determination is the most trustworthy parameter [60].

Methanol is a universal solvent in the extraction of phytochemicals for investigation purposes [61]. For extracting plant components that are soluble in water (polysaccharides, polypeptides, and tannins), mixtures of solvents are quite common; the most frequent ones being alcohol and water. This mixture could also extract most of the polar and nonpolar compounds of the plant. Thus, in this study, 80% methanol was preferred to serve as a solvent mixture. Using 80% methanol for extraction of *G. ternifolia* was also justified based on the previous studies that alcohol would be a better solvent for extraction of this plant [34]. To repeat the ethnomedicinal administration method and the possible route during clinical assessment, the oral route was opted in the present study to administer the hydromethanolic extract and solvent fractions of the plant [57]. Regarding the acute oral toxicity test, a hydroalcoholic stem bark extract of *G. ternifolia* did not cause any observable damage in the study mice at 2,000 mg/kg. Earlier reports have shown that if the LD_50_ value of a test chemical is 3 times more than the minimum effective dose, the extract is taken as a good candidate for further study [62, 63]. This could enlighten the safe use of the study plant to manage malaria by the local community in Ethiopia.

The antimalarial properties of the crude extract and fractions of the stem barks of *G. ternifolia* were assessed using three models. From the results, one could see that the % parasitemia measured in the four-day test was reduced by the hydroalcoholic extract in infected mice. The parasite suppression exhibited was comparable with *Terminalia brownie* [18]. Regarding fractions, n-butanol and chloroform fractions produced higher chemosuppressive results than an aqueous fraction, indicating the localization of active constituents in these two fractions. The parasite suppressive effects of *G. ternifolia* might be through the indirect improvement of the immune system or by suppressing/blocking other target pathways, which are not fully realized [64]. The steroids, flavonoids, and saponins noticed in *G. ternifolia* stem barks have been proved to possess significant immunomodulatory effects. The therapeutic benefits of traditional remedies are often ascribed to the availability of bioactive constituents [65–67].

High levels of chemosuppression (schizonticidal activity) were produced at higher doses of the hydromethanolic extract and its solvent fractions, indicating the increment of active metabolites as the dose increases. This finding agrees with a previous *in vitro* study carried on the same plant that the extracts and isolated compounds were lethal at high concentrations but inhibited growth at low concentrations. Maximum parasitemia suppression (57.84%) was obtained by the crude extract of the study plant compared with its fractions in the 4-day suppressive test. This is in line with the previous investigation that the crude extracts of *G. ternifolia* showed a significant *in vitro* activity against *P. falciparum* compared with the fractions possibly due to the synergistic effect of flavonoids components [44].

The highest effect by n-butanol fraction next to the crude extract might have resulted from the presence of the same phytoconstituents in these test drugs except the absence of terpenoids in the n-butanol fraction (Table 7). The reason behind a little bit closer effect to the extract by n-butanol fraction might have also been instigated from the alcoholic nature of the two solvents. This finding is also consistent with other experiments in which the n-butanol fraction had greater activity than chloroform and water fractions [68, 69]. The chloroform fraction lacks detectable anthraquinone (Table 7), which probably explains why it had lower activity than the n-butanol fraction. The results of chloroform fraction are comparable with the effect of the chloroform fraction of *Dodonaea angustifolia* and *Vernonia amygdalina* reported in other studies [70, 71]. However, all doses of the aqueous fraction and the lowest dose of both n-butanol and chloroform fractions were unable to exhibit antimalarial activity (to be active, the extract must suppress percentage parasitemia by greater than 30%) [72]. This effect might be due to the absence of sufficient concentration of active constituent(s) or related to loss of some active ingredient(s) due to inadequate physiological uptake of the test dose. Better activity reduction in the solvent fractions than that of the crude extract could be justified by the loss of synergistic activity among the compounds or differential distribution of secondary metabolites within the fractions. This result is in agreement with other findings [60, 73]. In fact, the very limited activity of the aqueous fraction might be attributed to the absence (presence in undetectable) of flavonoids, steroids, and tannins in this fraction (Table 7). The antiplasmodial activity of these phenolic compounds (flavonoids and tannins) detected from *G. ternifolia* is confirmed in previous studies done on the same plant [44, 74].

As it has been shown in the result that the crude extract exerted higher parasitemia suppression during the four-day suppressive study, its curative outcome on established infection was further assessed using Rane's test model. The hydroalcoholic extract of stem barks of *G. ternifolia* showed significant suppression of parasitemia. This result is in comparison with the curative effect of *Piliostigma thonningii* root bark extract [63]. It is most likely to consider this study plant as a possible source for new antiplasmodial agents as it is promising to have both suppressive and curative effects by a phytodrug [75]. The parasitemia inhibition activity in the curative test was superior to the activity in the four-day suppressive test, which is in line with other findings [76, 77], most likely due to the nonselective activity of the extract against the proliferative processes of the malaria parasite. The entry of the parasite into the blood alone does not produce disease, but the reactivity of the host immune system against foreign pathogenic microbes through free radical generation, phospholipase cascade activation, and production of prostaglandins and other hemolytic principles including free fatty acids are also involved [76]. Thus, the prominent antiplasmodial activity observed on the curative model may be due to the suppressive effect of the crude extract on free radical and hemolytic factor generation by the parasite [78]. This study plant has been scientifically validated for its beneficial effects as antiaggregating/antipolymerization and free radical scavenging ability [79]. These mechanisms of action could be evidenced by the *in vitro* study in which the flavonoid aglycones isolated from *G. ternifolia* leaves have antioxidant nature that neutralizes the oxidative damage induced by the *Plasmodium* species [80]. In the present study, the stem bark of *G. ternifolia* was shown to have flavonoids. Moreover, the antioxidant property of saponins, phenols, and tannins, detected in this study, was reported in previous studies done on different plants [81, 82]. In advance, there is a suggestion that extracts of *G. ternifolia* have a similar manner of action to that of mefloquine and chloroquine [43]. On the other hand, the curative potential of the crude extract could be attributed to terpenoids and flavonoids found within the extract, respectively, through inhibition of protein synthesis and chelation with the nucleic acid base pairing of the parasite [83].

Since the curative effect of the study plant was promising, the chemoprophylactic potential of the hydromethanolic extract was further investigated by repository tests. The output of this test indicates that the plant extract induced a preventive effect against parasitemia proliferation in a dose-dependent manner. Although the hydroalcoholic extract significantly suppressed parasitemia, it was smaller than the effect observed in four-day suppressive and Rane's tests. Similar outcomes in which plants possessed enhanced suppressive and curative effects than chemoprophylactic effects were stated in other research studies [76, 77, 84]. This might be due to the administration of the extract prior to infection establishment, i.e., it might be rapidly metabolized and/or excreted [85]. It can also be due to the model we used (lacks the insect vector), the inoculation manner, and the doses used that result in rapid RBC infection without the parasite passing through the hepatic stages [57]. The other likelihood was that the extract might act by the metabolic activation of the immune system. As a result, the removal of the parasite could not be total [50]. The result here was, however, contrary to other investigations where plants had higher residual activity than suppressive and curative activity [86].

In the current study, the crude extract of stem barks of *G. ternifolia* prolonged survival days on early infection, which is consistent with *T. brownie* [18]. In line with *Croton macrostachyus* [60], the crude extract produced a greater survival time than the solvent fractions. From the solvent fractions, the two higher doses of both chloroform and n-butanol fractions significantly improved the survival date of the experimental mice in early infection. This is consistent with *D. angustifolia* and *V. amygdalina* [70, 71]. Parasitemia reduction induced by the crude extract observed in Rane's test is translated into a longer survival time. Except for the lowest dose, the crude extract of stem barks of *G. ternifolia* significantly increased the MST in the chemoprotective test compared with the placebo treatment. The prolongation effect in MST at all three tests could be directly associated with the low parasite level and the overall improvement in pathologic effect imposed by the test doses [49, 68]. This effect is additional evidence regarding the antimalarial efficacy of the plant extract. However, the mean survival days of the mice treated with chloroquine was appreciably increased compared with all the hydroalcoholic extract-treated groups in all the models; this might be due to the rapid clearance phase or less potency of the extracts [21].

Experimental drugs active against *P. berghei* were known for antimalarial activities [78]. Therefore; we could say that stem barks of *G. ternifolia* possessed antimalarial activity. This assertion is supported by *in vivo* experiments that confirmed antiplasmodial effects of root barks and leaves of *G. ternifolia* [87, 88] and other species of the same genus such as *Gardenia lutea* and *Gardenia sokotensis* [73, 89]. Drugs having antibacterial activity such as doxycycline and clindamycin are used in the therapy of malaria. Concerning this, the current study plant (*G. ternifolia*) has *in vitro* antimicrobial activities [37, 79]. This finding further supports the notion that the study samples have antiplasmodial activity. In addition, this study plant has *in vitro* antiparasitic action against *Theileria lestoquardi* (an apicomplexan parasite that infects RBCs) [32]. Hence, the similarity of this ruminant parasite to the human parasite *Plasmodium* both in pathogenesis and phylum taxonomy [90] asserted the antiplasmodial activity of stem barks of *G. ternifolia*.

As revealed in the preliminary phytochemical screening, the crude extract and solvent fractions of the stem barks of *G. ternifolia* are rich in various secondary metabolites including alkaloids, flavonoids, saponins, steroids, tannins, and terpenoids. This is in agreement with the past studies done on *G. ternifolia* that its root barks and leaves contain alkaloids, anthraquinones, flavonoids, phenols, saponins, sterols, tannins, and terpenoids [87, 88]. In association with this, the bioactive compounds, five flavonoids (quercetin-4; 7-O-dimethyl ether; kaempferol-7-O-methyl ether; naringenin-7-O-methyl ether; and 4,5-dihydroxy-6,7-dimethoxyflavanone), and two steroids (*β*-sitosterol and stigmasterol) isolated from the aerial parts of *G. ternifolia* in other studies are effective against both chloroquine-sensitive and chloroquine-resistant strains of *P. falciparum* [44]. Furthermore, the antiplasmodial activity of the plant in the current study might be attributed to the two glycoflavonoid compounds (stachannin and pectolinarigenin-7-O-glucoside) and two phenolics (acteoside and isoacteoside) previously isolated from stem barks of *G. ternifolia* [74]. Thus, based on the witness from the past ethnobotanical and *in vitro* studies as well as from the current study, the antiplasmodial activity of stem barks of *G. ternifolia* could be ascribed to the secondary metabolites present in our experimental plant.

Biologically active chemicals present in the hydroalcoholic extract and its fractions of the study plant could induce antiplasmodial activity through different modes of action. Phytochemicals like anthraquinones cause intercalation in DNA [91]. The phytoconstituents such as saponins can form complexes with cholesterol in biological membranes and bind to surface glycolipids and glycoproteins [16]. Besides, flavonoids and steroids can inhibit the multiplication and growth of the *Plasmodium* species by blocking the influx of important nutrients that are necessary for their survival [19]. Flavonoids also have the capacity to make a complex with soluble and extracellular proteins and to make a complex with cellular components of the *Plasmodium* parasite. Flavonoids with high lipophilic nature may disrupt the malaria parasite membranes, inactivate toxins, and inhibit some enzymes [44, 92]. Moreover, tannins may stimulate phagocytic cells and host-mediated tumor activity complexation with proteins [93]. Cytotoxicity is another mechanism exerted by phytochemicals such as alkaloids and terpenoids [19]. Alkaloids are known for the ability that they intercalate with DNA and terminate cell division [94].

Hypoglycemia and reduction in body weight, body temperature, and PCV are cardinal signs of malaria-infected mice [95]. Extracts of an ideal medicinal plant with antiplasmodial activity are expected to avert a malaria-caused decline in body weight, temperature, and PCV due to the increase in parasitemia. Like quinine and proguanil antimalarial drugs, the current study plant has antihyperglycemic tendency in mice, which might make it the preferred drug to treat malaria patients with diabetes mellitus [96].

Body weight loss protection by the 600 mg/kg dose of the extract of stem barks of *G. ternifolia* on early infection in the present study was significant compared with the placebo drug. This might have been determined by nutritional components of the plant than other detrimental factors [32]. The two higher doses of n-butanol fraction and the largest dose of aqueous fraction showed a remarkable increase in body weight. This finding might have been contributed by the improvement in packed cell volume, rectal temperature, and removal of parasites among treated mice as shown in the result section. Vitamins and minerals present in fruit pulps of *G. ternifolia* [97] could also be present in stem barks and might contribute to weight increment in treated mice by enhancing food intake capacity. However, parasite suppression by all doses of chloroform fraction and lower doses of n-butanol and aqueous fractions was not translated into the protection of loss in body weight. This might be due to the consequence of metabolic malfunction and hypoglycemia related to malaria infection [49]. Better than the activities demonstrated in the four-day suppressive study, the 80% hydromethanolic extract protected weight loss (at higher doses) and a drop in body temperature at the curative test. This could be ascribed to the greater parasitemia suppression and survival date prolongation in Rane's test. In residual infection, only the 600 mg/kg dose of the crude extract improved body weight.

A decrease in the metabolic rate of infected mice occurs before death and is accompanied by a corresponding decrease in internal body temperature contrary to the situation in human subjects [62, 68]. In the four-day suppressive test, the highest dose of the hydroalcoholic extract averted temperature drop significantly compared with the vehicle (negative control). The 400 and 600 mg/kg doses of chloroform, n-butanol, and aqueous fractions have also protected the drop in body temperature in treated mice. The changes observed with regard to temperature were correlated to body weight changes measured during the experiment rather than parasite suppression, indicating that weight loss can be indirectly influenced by malaria fever [98]. The crude extract protected the decrease in rectal temperature in malaria-infected mice at every dose in the curative test but at a higher dose in the prophylactic test. The protective effect observed on residual infection is in line with the largest dose effect by *Syzygium guineense* [99]. Those protective activities might be due to the amelioration of some pathologic conditions and modulation of the immune system by secondary metabolites like steroids, saponins, anthocyanins, and flavonoids [68].

In the chemosuppressive study, the largest dose of the hydroalcoholic extract, as well as the higher doses of chloroform and n-butanol fractions of stem barks of *G. ternifolia*, prevented the fall in PCV significantly compared with the respective placebo agent. When the concern was an increase in activity as dose too, the effect produced is in line with *C. macrostachyus* and *Ajuga integrifolia* [60, 69]. This protective effect might have resulted from the significant parasitemia suppression brought by the active constituent(s) in the given doses of the extract because the rise in blood parameters is usually associated with the decrease in parasite load [100]. Anthocyanins and organic acids identified in other studies [79] from this plant could also be responsible for the protection of anemia because these metabolites have the capability to interact with proteins and stabilize the RBC membrane by protecting the oxidation of membrane phospholipids [101]. These could counteract hemolysis associated with saponins caused by increasing the permeability of the RBC plasma membrane [102]. Species of this plant are also known for their antidehydrating properties, which further protect PCV reduction in infected mice [79]. Opposite to this effect, the crude extract did not prevent PCV decline both at curative and prophylactic tests. This discrepancy could be ascribed to the time difference in the administration of the test samples between the three models.

## 5. Conclusion

Generally, the current results showed that 80% methanolic extract and its solvent fractions of *G. ternifolia* stem barks have antiplasmodial activities. The findings confirmed that the study plant has a high curative effect. N-butanol and chloroform fractions were found to be active among the fractions, indicating that the semipolar and nonpolar compounds of the plant are responsible for the antimalarial effects of *G. ternifolia*. The output of the current study would offer evidence to support the previous *in vitro* research on stem barks, root barks, and leaf surface exudates of the plant as well as the assertion made by the Ethiopian traditional medical healers.

## Figures and Tables

**Table 1:** Percentage parasitemia and mean survival days of malaria-infected mice treated with the hydroalcoholic extract and solvent fractions of stem barks of *G. ternifolia* in the four-day suppressive test.

Groups	% parasitemia	% suppression	Survival time
DW	43.64 ± 2.17	0.00	6.92 ± 0.08
200 mg/kg CE	29.60 ± 2.93	32.16^a∗∗∗b∗∗∗e∗∗∗^	8.00 ± 0.63^b∗∗∗d∗e∗∗∗^
400 mg/kg CE	23.74 ± 0.90	45.59^a∗∗∗b∗∗∗^	10.25 ± 0.51^a∗∗∗b∗∗∗e∗^
600 mg/kg CE	18.39 ± 0.77	57.84^a∗∗∗b∗∗∗^	12.42 ± 0.58^a∗∗∗b∗∗∗^
25 mg/kg CQ	0.00 ± 0.00	100.00^a∗∗∗^	28.00 ± 0.00^a∗∗∗^

2% TW80	46.83 ± 2.90	0.00	7.00 ± 0.26
200 mg/kg CF	36.27 ± 0.68	22.54^a∗∗∗b∗∗∗e∗∗^	7.42 ± 0.33^b∗∗∗e∗∗∗^
400 mg/kg CF	32.15 ± 1.94	31.34^a∗∗∗b∗∗∗^	8.25 ± 0.38^a∗b∗∗∗e∗∗^
600 mg/kg CF	28.74 ± 1.70	38.62^a∗∗∗b∗∗∗^	9.92 ± 0.20^a∗∗∗b∗∗∗^
25 mg/kg CQ	0.00 ± 0.00	100.00^a∗∗∗^	28.00 ± 0.00^a∗∗∗^

DW	48.51 ± 1.30	0.00	6.92 ± 0.24
200 mg/kg BF	34.23 ± 1.11	29.44^a∗∗∗b∗∗∗d∗∗∗e∗∗∗^	7.92 ± 0.27^b∗∗∗d∗∗∗e∗∗∗^
400 mg/kg BF	27.57 ± 1.36	43.17^a∗∗∗b∗∗∗^	10.00 ± 0.43^a∗∗∗b∗∗∗e∗^
600 mg/kg BF	24.75 ± 0.54	48.98^a∗∗∗b∗∗∗^	11.50 ± 0.37^a∗∗∗b∗∗∗^
25 mg/kg CQ	0.00 ± 0.00	100.00^a∗∗∗^	28.00 ± 0.00^a∗∗∗^

DW	44.94 ± 2.54	0.00	7.17 ± 0.11
200 mg/kg AF	38.85 ± 0.52	13.56^a∗∗∗b∗∗∗d∗e∗∗∗^	7.25 ± 0.28^b∗∗∗^
400 mg/kg AF	36.56 ± 0.86	18.65^a∗∗∗b∗∗∗e∗^	7.67 ± 0.51^b∗∗∗^
600 mg/kg AF	34.24 ± 0.67	23.81^a∗∗∗b∗∗∗^	7.92 ± 0.27^b∗∗∗^
25 mg/kg CQ	0.00 ± 0.00	100.00^a∗∗∗^	28.00 ± 0.00^a∗∗∗^

Data are expressed as mean ± SEM (*n* = 6); a, compared with DW; b, compared with positive control; d, compared with 400 mg/kg; e, 600 mg/kg; ^*∗*^*P* < 0.05; ^*∗∗*^*P* < 0.01; ^*∗∗∗*^*P* < 0.001; DW for distilled (pure) water (negative control); CE for crude extract; 2% TW80 for 2% Tween-80; CF for chloroform fraction; BF for n-butanol fraction; AF for aqueous (water) fraction; and CQ for chloroquine (positive control).

**Table 2:** Body weight and rectal temperature of *P. berghei*-infected mice treated with the crude extract and solvent fractions of stem barks of *G. ternifolia* in the 4-day suppressive test.

Groups	Body weight (g)			Rectal temperature (°C)		
	D0	D4	% change	D0	D4	% change
DW	30.85 ± 0.99	29.25 ± 1.14	−5.28	37.15 ± 0.25	34.80 ± 0.63	−6.31
200 mg/kg CE	31.08 ± 2.32	30.10 ± 2.02	−2.88^b∗∗^	37.00 ± 0.16	35.42 ± 0.49	−4.28^b∗^
400 mg/kg CE	32.10 ± 2.24	31.28 ± 2.25	−2.59^b∗∗^	37.25 ± 0.20	36.30 ± 0.32	−2.56
600 mg/kg CE	32.00 ± 1.85	31.45 ± 1.76	−1.66^a∗b∗^	37.35 ± 0.29	37.16 ± 0.18	−0.49^a∗^
25 mg/kg CQ	31.70 ± 1.16	32.43 ± 1.11	2.36^a∗∗∗^	36.80 ± 0.30	37.15 ± 0.46	0.98^a∗∗^

2% TW80	26.40 ± 0.96	23.75 ± 0.90	−9.99	37.90 ± 0.19	35.15 ± 0.33	−7.26
200 mg/kg CF	26.50 ± 0.79	24.30 ± 0.52	−7.96^b∗∗^	36.95 ± 0.17	34.70 ± 0.25	−6.08^b∗∗∗e∗∗^
400 mg/kg CF	27.05 ± 0.74	25.80 ± 0.65	−4.57^b∗^	37.75 ± 0.16	36.20 ± 0.09	−4.10^a∗∗b∗∗∗^
600 mg/kg CF	26.25 ± 0.77	25.73 ± 1.22	−2.19	37.65 ± 0.15	36.50 ± 0.19	−3.05^a∗∗∗b∗∗∗^
25 mg/kg CQ	28.30 ± 0.72	29.90 ± 0.58	5.94^a∗∗^	37.55 ± 0.23	38.08 ± 0.17	1.43^a∗∗∗^

DW	27.77 ± 0.98	24.72 ± 0.95	−11.02	36.90 ± 0.21	34.65 ± 0.14	−6.08
200 mg/kg BF	28.70 ± 0.58	26.43 ± 0.53	−7.85^b∗∗∗e∗∗^	37.17 ± 0.27	35.67 ± 0.51	−4.05^b∗∗∗^
400 mg/kg BF	27.32 ± 0.91	26.20 ± 0.69	−3.98^a∗∗b∗∗^	37.57 ± 0.17	36.42 ± 0.17	−3.06^a∗b∗∗∗^
600 mg/kg BF	28.10 ± 0.69	27.80 ± 0.48	−0.97^a∗∗∗^	36.92 ± 0.32	36.25 ± 0.28	−1.79^a∗∗∗b∗∗^
25 mg/kg CQ	29.15 ± 0.56	30.12 ± 0.47	3.40^a∗∗∗^	37.40 ± 0.24	38.05 ± 0.19	1.75^a∗∗∗^

DW	27.00 ± 0.95	24.18 ± 0.75	−10.35	37.20 ± 0.32	34.05 ± 0.34	−8.46
200 mg/kg AF	28.75 ± 0.54	26.35 ± 0.48	−8.32^b∗∗∗^	37.15 ± 0.18	34.48 ± 0.44	−7.19^b∗∗∗e∗∗^
400 mg/kg AF	29.28 ± 0.83	27.10 ± 0.91	−7.51^b∗∗∗^	36.80 ± 0.31	34.67 ± 0.22	−5.78^a∗∗b∗∗∗^
600 mg/kg AF	26.92 ± 0.31	25.42 ± 0.35	−5.57^a∗∗b∗∗∗^	37.30 ± 0.20	35.72 ± 0.15	−4.24^a∗∗∗b∗∗∗^
25 mg/kg CQ	28.72 ± 0.69	30.30 ± 0.74	5.52^a∗∗∗^	37.50 ± 0.12	37.93 ± 0.16	1.16^a∗∗∗^

Data are expressed as mean ± SEM (*n* = 6); a, compared with DW; b, compared with positive control; d, compared with 400 mg/kg; e, 600 mg/kg; ^*∗*^*P* < 0.05; ^*∗∗*^*P* < 0.01; ^*∗∗∗*^*P* < 0.001; DW for distilled (pure) water (negative control); CE for crude extract; 2% TW80 for 2% Tween-80; CF for chloroform fraction; BF for n-butanol fraction; AF for aqueous (water) fraction; and CQ for chloroquine (positive control); D0 for pre-treatment value on day 0; D4 for post-treatment value on day 4.

**Table 3:** Percentage parasitemia and mean survival days of malaria-infected mice treated with the hydroalcoholic extract and solvent fractions of *G. ternifolia* stem barks in Rane's test.

Groups	% parasitemia	% suppression	Survival time
DW	53.09 ± 1.34	0.00	7.08 ± 0.27
200 mg/kg CE	33.87 ± 1.52	36.22^a∗∗∗b∗∗∗d∗∗∗e∗∗∗^	9.00 ± 0.22^b∗∗∗d∗e∗∗∗^
400 mg/kg CE	26.13 ± 1.65	50.79^a∗∗∗b∗∗∗e∗∗^	11.17 ± 0.38^a∗∗∗b∗∗∗e∗∗^
600 mg/kg CE	20.33 ± 0.58	61.72^a∗∗∗b∗∗∗^	13.92 ± 0.92^a∗∗∗b∗∗∗^
25 mg/kg CQ	0.00 ± 0.00	100.00^a∗∗∗^	28.00 ± 0.00^a∗∗∗^

Data are expressed as mean ± SEM (*n* = 6); a, compared with DW; b, compared with positive control; d, compared with 400 mg/kg; e, 600 mg/kg; ^*∗*^*P* < 0.05; ^*∗∗*^*P* < 0.01; ^*∗∗∗*^*P* < 0.001; DW for distilled water (negative control); CE for crude extract; CQ for chloroquine (positive control).

**Table 4:** Body weight, rectal temperature, and packed cell volume of malaria-infected mice treated with the hydroalcoholic extract of stem barks of *G. ternifolia* in Rane's test.

Groups	Body weight (g)			Temperature (°C)			Packed cell volume		
	D3	D7	% change	D3	D7	% change	D3	D7	% change
DW	32.67 ± 0.82	29.04 ± 1.01	−11.05	37.05 ± 0.14	32.10 ± 0.52	−13.36	43.33 ± 1.61	38.64 ± 1.71	−10.76
200 mg/kg CE	32.50 ± 0.96	29.93 ± 0.98	−7.91^b∗^	37.72 ± 0.13	34.35 ± 0.31	−8.92^a∗∗b∗∗∗e∗∗^	46.00 ± 2.42	42.41 ± 2.49	−7.79
400 mg/kg CE	32.57 ± 1.08	31.11 ± 1.11	−4.52^a∗^	37.85 ± 0.24	35.40 ± 0.39	−6.46^a∗∗∗b∗∗∗^	53.25 ± 1.17	51.15 ± 1.94	−4.08
600 mg/kg CE	32.84 ± 0.65	31.96 ± 0.59	−2.65^a∗∗^	37.48 ± 0.26	36.10 ± 0.44	−3.70^a∗∗∗b∗∗^	45.00 ± 1.83	43.77 ± 1.18	−2.44
25 mg/kg CQ	32.43 ± 1.01	31.94 ± 1.21	−1.62^a∗∗^	37.00 ± 0.17	37.33 ± 0.22	0.91^a∗∗∗^	47.35 ± 0.89	48.35 ± 1.02	2.11^a∗^

Results are expressed as mean ± SEM (*n* = 6); a, compared with DW; b, compared with positive control; d, compared with 400 mg/kg; e, 600 mg/kg; ^*∗*^*P* < 0.05; ^*∗∗*^*P* < 0.01; ^*∗∗∗*^*P* < 0.001; DW for distilled (pure) water (negative control); CE for crude extract; CQ for chloroquine (positive control); D3 for pre-treatment value on day 3; D7 for post-treatment value on day 7.

**Table 5:** Percentage parasitemia and mean survival days of malaria-infected mice treated with the crude extract and solvent fractions of stem barks of *G. ternifolia* in the repository test.

Groups	% parasitemia	% suppression	Survival time
DW	26.69 ± 0.93	0.00	7.50 ± 0.18
200 mg/kg CE	21.15 ± 0.44	20.76^a∗∗∗b∗∗∗d∗e∗∗∗^	7.92 ± 0.15^b∗∗∗e∗∗^
400 mg/kg CE	19.31 ± 0.39	27.67^a∗∗∗b∗∗∗^	8.42 ± 0.20^a∗b∗∗∗^
600 mg/kg CE	17.77 ± 0.69	33.44^a∗∗∗b∗∗∗^	9.00 ± 0.13^a∗∗∗b∗∗∗^
25 mg/kg CQ	2.26 ± 0.24	91.53^a∗∗∗^	18.75 ± 0.31^a∗∗∗^

Results are expressed as mean ± SEM (*n* = 6); a, compared with DW; b, compared with positive control; d, compared with 400 mg/kg; e, 600 mg/kg; ^*∗*^*P* < 0.05; ^*∗∗*^*P* < 0.01; ^*∗∗∗*^*P* < 0.001; DW for distilled (pure) water (negative control); CE for crude extract; and CQ for chloroquine (positive control).

**Table 6:** Body weight, rectal temperature, and packed cell volume of malaria-infected mice treated with the hydroalcoholic extract of stem barks of *G. ternifolia* in the repository test.

Groups	Body weight (g)			Temperature (°C)			Packed cell volume		
	D3	D7	% change	D3	D7	% change	D3	D7	% change
DW	28.60 ± 1.57	26.75 ± 1.31	−6.30	36.95 ± 0.26	34.98 ± 0.35	−5.32	53.30 ± 1.44	50.77 ± 1.55	−4.69
200 mg/kg CE	29.55 ± 1.22	27.93 ± 1.22	−5.52^b∗∗∗e∗∗^	37.05 ± 0.27	35.55 ± 0.45	−4.06^b∗∗e∗^	57.00 ± 1.17	54.85 ± 1.24	−3.73
400 mg/kg CE	30.60 ± 2.36	29.45 ± 2.31	−3.81^b∗∗∗^	37.22 ± 0.34	36.10 ± 0.34	−2.94	55.60 ± 1.56	53.95 ± 1.23	−2.88
600 mg/kg CE	27.40 ± 1.13	26.85 ± 1.13	−2.02^a∗∗^	36.80 ± 0.27	36.33 ± 0.27	−1.26^a∗^	54.10 ± 1.81	53.43 ± 1.89	−1.26
25 mg/kg CQ	31.65 ± 1.90	31.90 ± 2.02	0.68^a∗∗∗^	37.25 ± 0.23	37.08 ± 0.14	−0.43^a∗∗^	56.20 ± 1.24	56.13 ± 1.22	−0.11

Results are expressed as mean ± SEM (*n* = 6); a, compared with DW; b, compared with positive control; d, compared with 400 mg/kg; e, 600 mg/kg; ^*∗*^*P* < 0.05; ^*∗∗*^*P* < 0.01; ^*∗∗∗*^*P* < 0.001; DW for distilled (pure) water (negative control); CE for crude extract; CQ for chloroquine (positive control); D3 for pre-infection value on day 3; D7 for postinfection value on day 7.

**Table 7:** Phytochemical screening of the hydromethanolic extract and fractions of stem barks of *G. ternifolia*.

Secondary metabolites	Crude extract	Solvent fractions		
		Chloroform fraction	Butanol fraction	Aqueous fraction
Alkaloids	+	+	+	−
Anthraquinone	+	−	+	+
Flavonoids	+	+	+	−
Saponins	+	+	+	+
Steroids	+	+	+	−
Tannins	+	+	+	−
Terpenoids	+	+	−	−

Note. + = presence; − = absence.

## Data Availability

Almost all of our study materials and data are included in the manuscript, and some of it will be made available to other researchers upon fair request.
